# Pneumorachis and pneumocephalus: Case report of a rare blunt chest trauma complication

**DOI:** 10.1016/j.amsu.2022.103349

**Published:** 2022-02-05

**Authors:** Ilyass Laaribi, Amine El Mouhib, Mohammed Amine Oulalit, Abdelilah El Rhalete, Houssam Bkiyar, Brahim Housni

**Affiliations:** a Department of Intensive Care Unit, Mohammed VI University Hospital, Oujda, Morocco; b Faculty of Medicine and Pharmacy, Mohammed First University, Oujda, Morocco; c Mohammed First University Oujda, FMP Oujda, LAMCESM, Oujda, Morocco

**Keywords:** Pneumorachis, Pneumocephalus, Pneumothorax, Blunt chest trauma

## Abstract

**Introduction:**

Pneumorachis, air in the spinal canal, is very rare and its association with pneumocephalus following blunt thoracic trauma remains exceptionally uncommon.

**Case presentation:**

We present the case of a 65-year-old patient, a pedestrian hit by a car driving at very high speed. The lesion assessment on admission showed a bilateral hemothorax of moderate abundance, a right pneumothorax of low abundance and a left pneumothorax of moderate abundance, subcutaneous cervico-dorsal emphysema, pneumocephalus and significant pneumorachis at the cervico-dorsal level without fracture of the base of the skull, sinuses or the spine.

**Discussion:**

We discuss the different etiologies of pneumorachis, the main hypotheses of the constitution of this air effusion and the principles of management.

**Conclusion:**

Pneumorachis associated with pneumocephalus in a traumatic context without bone lesions is an extremely rare entity, its discovery should lead to further investigations to look for any spinal or basilar skull fracturethat could expose to an infectious risk or require a surgical procedure.

## Highlights

• The pneumorrachis was first described in 1977.
• Pneumorachis is mainly reported as an iatrogenic complication, and more rarely traumatic.
• Pneumorachis associated with pneumocephalus in a traumatic context without bone lesions is an extremely rare entity.

## 1 Introduction

Pneumorachis, air in the spinal canal, is a very rare entity first described in 1977 [1]. Pneumorachis is mainly reported as an iatrogenic complication, and more rarely traumatic, indeed, rare are the cases which have been cited in the literature [2], These cases are even more rare in blunt chest trauma [3,4]. However, to our knowledge, only one publication mentions its association with pneumocephalus following closed thoracic trauma without fracture of the skull base, sinuses or the spine [5].

We report in this article a case of pneumorachis associated with pneumocephalus, after a severe blunt chest trauma.

## 2 Case presentation

A 65-year-old man, with no previous medical history, presented to the emergency room with significant trauma and brief loss of consciousness following a high speed motor vehicle accident, where he was pedestrian.

The clinical examination on arrival revealed an agitated patient, with Glasgow Coma Score of 11/15, without sensory-motor deficit, exquisite pain on palpation of the cervico-dorsal spinous processes. On the respiratory plan, he had an arterial oxygen saturation of 84% inambient air, he was polypneic at 24 breaths per minute; Pulmonary auscultation found decreased vesicular murmur in both pulmonary fields. Hemodynamically; Blood pressure was at 75/32 mmHg, heart rate of 135 beats/min. There were no external injuries on the chest or neck. The rest of the examination revealed a deformity of both lower limbs associated with ecchymosis and multiple abrasions.

The initial management consisted of oxygen therapy with a high concentration mask, a vascular filling, vasopressor support and analgesic treatment. Shortly, after his admission the patient has become agitated and altered his consciousness prompting his intubation after rapid sequence induction.

Arterial blood gases under FIO2 = 40% were as follows: pH: 7.34; PaO2: 106 mmHg; PaCO2: 32 mmHg; HCO3: 19 mmol/l; SaO2: 98%. Lactate: 3.2 mmol/l.

The blood count showed a hemoglobin concentration of 8.7 g/dl, hemostasis and blood ionogram were normal.

**Fig. 1:**
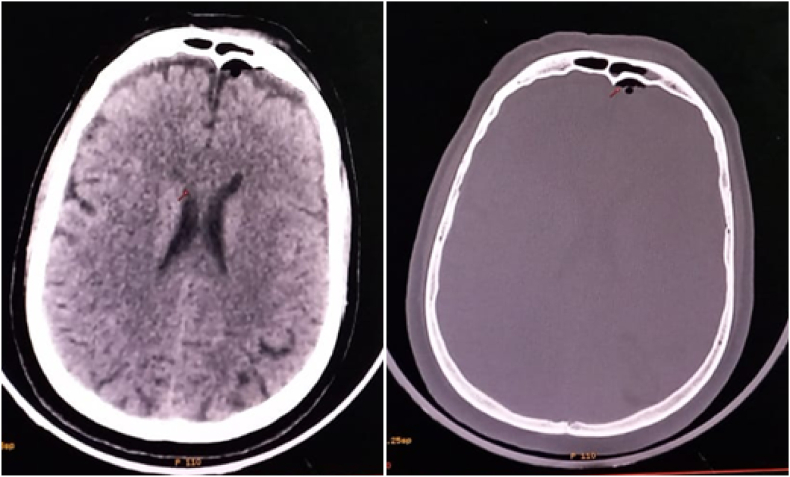
Brain computed tomography, showing frontal pneumocephalus and meningeal hemorrhage.

**Fig. 2:**
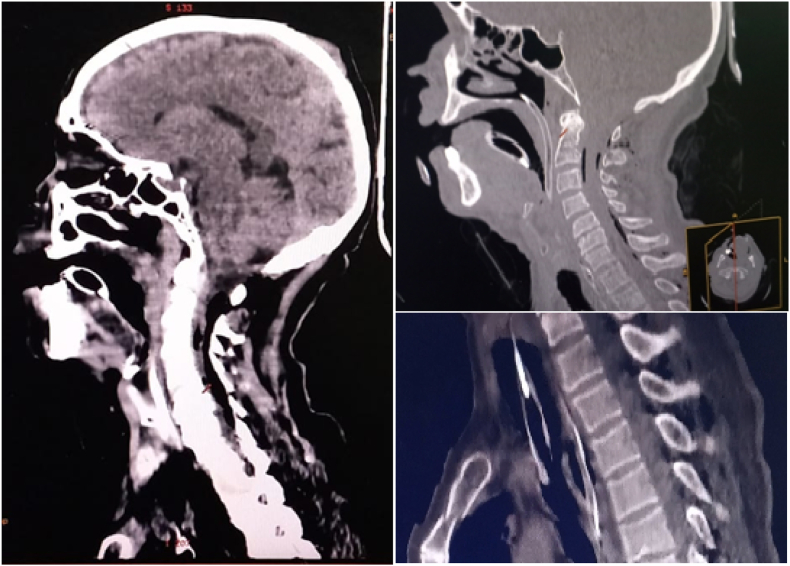
Saggital cervical and dorsal spine tomography showing pneumorachis on multiple levels.

**Fig. 3:**
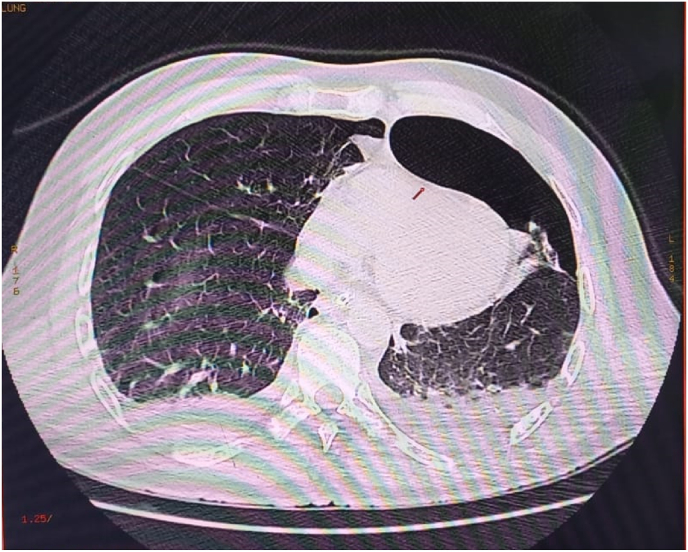
Axial sequence of a lung window chest computed tomography revealing a left pneumothorax with bilateral hemothorax.

After stabilization of the patient, computed tomography (CT) scan examinations were performed: Brain CT revealed a pneumencephalus and meningeal hemorrhage without skull base or sinus fracture (Fig. 1). Thoracic CT showed a bilateral hemothorax, a right pneumothorax of low abundance and a left pneumothorax of moderate abundance that was drained (Fig. 2), subcutaneous cervico-dorsal emphysema and the presence of intradural air on several levels (Fig. 3). There was no abdominopelvic injury. The rest of radiological assessment found a comminuted fracture of the left femur and a fracture of the right tibia and fibula.

The symptomatic treatment was continued and the evolution was unfavorable marked by the installation of a hemorrhagic shock and disseminated intravascular coagulation leading to the death of the patient the day after his accident.

## 3 Discussion

The term pneumorachis refers to the presence of air in the spinal canal and is a very rare entity whose traumatic origin is exceptionally rare [6]. Pneumocephalus is widely known and is defined by an intracerebral accumulation of air which can be iatrogenic or post-traumatic [7].

The most predominant etiology of pneumorachis remains iatrogenic, whether it is a complication of epidural anesthesia, lumbar puncture or spinal surgery [8]. It can also be secondary to local gas production during epidural abscesses, which has been described in patients with diabetes or Crohn's disease [9]. Another non-traumatic etiology is air migration of extrinsic origin, for example in pneumomediastinum or pneumothorax [10].

Exceptionally, trauma can lead to pneumorachis, this situation has been described at the cervical level following an extension of pneumocephalus secondary to craniofacial trauma with fracture of the skull base or paranasal sinuses or in spinal trauma [[9], [11], [12]]. This mechanism does not seem to be involved in our observation because the cerebtal and spinal CT scan did not reveal any fracture.

Much more rarely, thoracic trauma with pneumothorax and/or pneumomediastinum can lead to pneumorachis [[3], [4], [5], [6], [7], [8], [9], [10], [11], [12], [13]]. But only one similar case of pneumocephalus and pneumorachis has been found in the literature, this extremely rare lesion mechanism has been mentioned in an observation having in common the presence of a left pneumothorax, a pneumorachis and apneumocephalus in the absence of skull base or sinus wall fracture. The hypothesis retained in this observation was the extension of the air from the thorax towards the spinal canal and then its diffusion towards the cranial cavity [5]. This also seems to be the case for our patient.

The diagnosis of pneumorachis is purely radiological and it may be done on CT scan by the presence of intracanal aerial clearness [11], the clinic remains unspecific, spontaneous resorption is the rule [14]. Only one publication reports the surgical treatment of postoperative pneumorachis [15]. In our case, the patient died one day after his admission from hemorrhagic shock and we could not control the resorption or persistence of pneumorachis and pneumocephalus.

## 4 Conclusion

The association of pneumorachis and pneumocephalus after a closed trauma of the thorax without associated bone injuries is possible but extremely rare. Clinicians must remain vigilant and investigate further to identify any spinal or basilar skull fracture that may expose to an infectious risk or require a surgical procedure.

This work is reported in line with the 2020 SCARE guidelines [16].

## Provenance and peer review

Not commissioned, externally peer reviewed.

## Conflicts of interest

The authors declare no conflict of interest.

## Sources of funding

This research was not funded

## Ethical approval

This is a case report that does not require a formal ethical committee approval. Data were anonymously registered in our database. Access to data was approved by the head of the department.

## Consent

Written informed consent was obtained from the patient for publication of this case report and accompanying images. A copy of the written consent is available for review by the Editor-in-Chief of this journal on request.

## Author contribution

Dr Ilyass Laaribi and Dr. Amine El Mouhib: are principal investigators that collected and analyzed data, wrote the manuscript and prepared the final draft for the submission. Prof. Brahim Housni and Prof. Houssam Bkiyar: supervised the research project and approved the final draft for publication All authors approved the final version of the manuscript.

## Registration of research studies

This is not an interventional study. We only reported the patient's findings from our database as a case series.

## Guarantor

Dr Ilyass Laaribi.
